# Predictive Genetic Testing in the Cancer Management and Prevention

**DOI:** 10.26502/jcsct.5079274

**Published:** 2025-10-27

**Authors:** Niayesh Najafi, Kayvan Sasaninia, Zoya Najafi, Cady Babakhan Vartanian, Kevin Babakhan Vartanian, Faredun Dungore, Devendra K. Agrawal

**Affiliations:** 1 Department of Translational Research, College of the Osteopathic Medicine of the Pacific, Western University of Health Sciences, Pomona, California, USA; 2 Department of Genetics and Molecular Biology, School of Medicine, Isfahan University of Medical Sciences, Isfahan, Iran; 3 Brainstim Centers, Calabasas, California

**Keywords:** Breast cancer, Cancer prevention, Colorectal cancer, Hereditary cancer, Ovarian cancer, Pancreatic cancer, Personalized medicine, Predictive genetic testing, Prostate cancer, Screening test, Uterine cancer

## Abstract

Predictive genetic testing is a medical test that can effectively reduce morbidity and mortality caused by genetic diseases. This specific test predicts the risk of disease and provides prognostic information in asymptomatic persons, leading to the adoption of prevention strategies and individualized treatment in high-risk persons. Although the cause of some cancers is not known correctly, numerous studies establish that some specific gene mutations make people susceptible to specific cancer. Therefore, genetic screening of people, especially individuals with a family history of cancer, can be essential in reducing disease mortality and improving survival. Although genetic screening, in some cases, is associated with challenges, overall, it can be recognized as an important way to prevent and control hereditary diseases and save many lives. This article provides a critical review of the published reports and discusses the value of preventive genetic testing in preventing and controlling cancers and the role of hereditary mutations associated with cancer development.

## Introduction

Predictive genetic testing uses a genetic test to predict the future disease risk in asymptomatic individuals, reducing morbidity and mortality through targeted screening and surveillance, and preventive strategies [1]. More generally, predictive genetic testing is a medical test that identifies mutation carriers, determines risk in asymptomatic individuals, provides a prognosis for clinical management, and predicts response to treatment and the future course of the disease in affected individuals, families, or groups [2, 3]. Cancer is a leading cause of death worldwide and the second leading cause of death in the United States. It was estimated that in 2022, around 1,918,030 new cancer cases and 609,360 cancer deaths would occur in the United States [4]. Mutations in high penetrate genes cause 5% of all bowel, breast, and ovary cancer cases and increase the lifetime cancer risk in Asymptomatic carriers up to 60–80% [5]. In addition to mutations in high penetrate genes that significantly increase cancer risk, genetic variations that convey modest changes in cancer risk and are characterized as low to moderate penetrant gene mutations can be the subject of predictive genetic testing [6]. Suppose a mutation in a person with the disease has been identified. In that case, predictive genetic testing can identify at-risk family members and adopt the best monitoring and treatment methods [5]. Besides, the identification of high-risk people by predictive genetic testing may lead to the adoption of an optimal screening strategy, including the shortening current screening interval for a specific condition in high-risk individuals (like colonoscopy in the case of colorectal cancer). This leads to decreasing the cost for persons and the health system [7]. Therefore, cancer Genetic screening, especially in cancers that do not have a good prognosis and diagnosis at the late stages, is critical to identifying high-risk individuals and provides the possibility of preventing and identifying cancer at the early stages [8]. Despite the success of preventive genetic testing in providing information for identifying, predicting, and prognosing heritable genetic diseases, this method has faced some issues. Several common challenges in using genetic tests are precise estimates of penetrance, time lag, prediction of polygenic disorders, variable expressivity, pleiotropy, uncertainties inherent, patient’s inability to report clinical information accurately, lack of physician’s recommendation, limitations of genetic testing in children, and reporting untreatable conditions [1, 3, 9, 10]. Studies demonstrate that although the medical implications of unwanted genomic information sadden patients and their families, most people are satisfied with this, and only a small percentage of people regret doing the test [11]. Furthermore, individuals with information about their genetic health often feel obligated to inform their close relatives and family members about their results, which facilitates conversations about preventive care and the health benefits of being aware of heritable genetic diseases [12, 13]. Overall, molecular genetic testing can play a significant role in individual risk prediction, leading to personalized treatment and prevention strategies that can be more effective than standard screening [14, 15]. This article reviews the importance of predictive genetic testing in some cancers and the role of several gene hereditary abnormalities in cancer development.

## Ovarian cancer

Ovarian cancer (OC) is the fourth leading cause of cancer-related death in women. Almost 10% of OCs are attributable to heritable mutations, and genetic counseling provides the opportunity to diagnose the mutation carriers [16]. Given the relatively low cost of genetic testing for OC, the demand for genetic tests is expected to increase. The hope underlying such testing is that early detection of people at risk can be done effectively in the direction of risk management and improvement of clinical results [17]. Generally, the significance of recognizing heritable mutations in women with OC extends beyond the primary treatment period, allowing cancer prevention and early detection for patients and their family members [18]. For instance, studies reveal that BRCA2 mutation predisposes women to hereditary breast and ovarian cancer. BRCA mutations cause approximately 10%–18% of OC cases [19, 20]. Table 1 highlights the selected studies from various countries providing information on the percentage of germline mutations in ovarian cancer [21–26].

**Figure 1:**
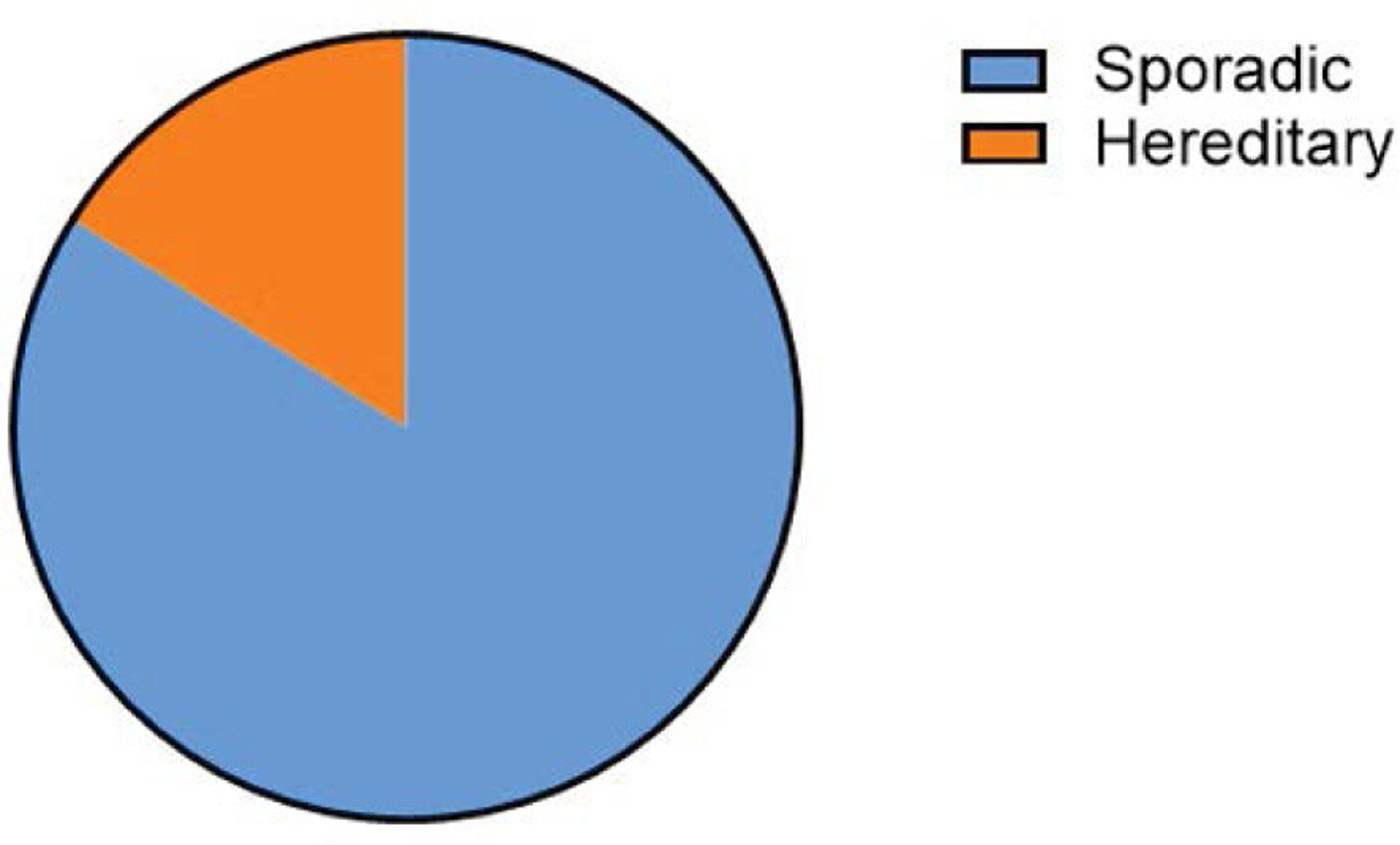
Pie Chart showing the distribution of sporadic and hereditary ovarian cancer.

Figure 1 shows that an average of 7.5% of ovarian cancers are hereditary, with the vast majority being sporadic (92.5%). This visualization highlights the importance of hereditary factors in ovarian cancer but also underscores that most cases arise sporadically [16,19].

**Figure 2:**
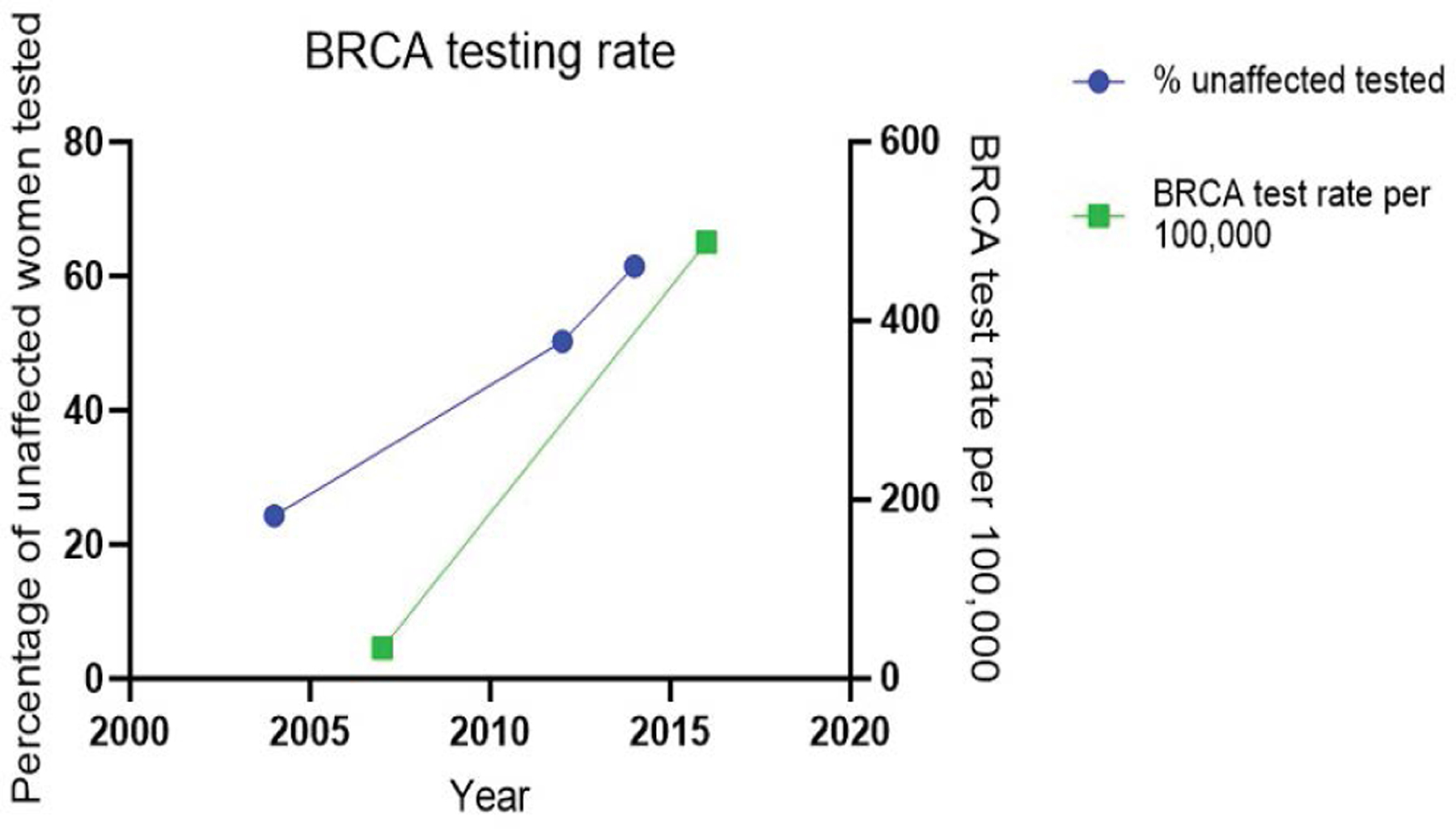
Increase in BRCA Testing Among Unaffected Women.

Figure 2 illustrates the significant increase in the proportion of BRCA tests performed on unaffected women over a decade, from 24.3% in 2004 to 61.5% in 2014 in the UK. BRCA test rate significantly increased from 34 per 100,000 women in 2007 to 488 per 100,000 women in 2016 in the United States. The trend line indicates a growing awareness and utilization of genetic testing among this group [16,19, 27]. This visualization helps to highlight the portion of ovarian cancer cases that could potentially benefit from targeted genetic testing and surveillance [16,28].

## Breast cancer

Breast cancer (BC) is among the most lethal cancers. This malignancy is estimated to affect about 12% of women over their lifetime [28]. For instance, about 330,000 BC cases are reported annually in the United States. Approximately 10% of BC and OC cases are caused by inheritable causes [29]. Recent studies demonstrate that factors related to increased estrogen levels during women’s lifetime and genetic variations related to familial histories, such as a family history of BC in first-degree relatives, predispose women to BC [30]. Therefore, genetic and non-genetic risk factors should be incorporated to improve screening programs for BC risk. People can be classified based on their risk of cancer incidence, leading to cost savings. In high-risk individuals, screening should be done more precisely using MRI, tumor segmentation, and mammography [30]. Criteria such as age at diagnosis, first-degree relatives (FDR) with BC (with an age of diagnosis), and FDR with OC are essential for detecting high-risk women [31]. Genetic testing has become increasingly applicable to systemic therapy [29]. For instance, BC/OC risks can be reduced by undergoing risk-reducing surgery, chemoprevention, or regular surveillance if a BRCA1/2 gene mutation carrier is identified. Additionally, genetic testing may save some women from unnecessary surgery and reduce healthcare costs [32]. These tests can also help determine the high-risk family members of affected persons through cascade family variant testing before the onset of cancer and facilitate the prevention of breast and other cancers [29]. The investigations reveal that a mutation in BRCA genes (BRCA1 or BRCA2) increases the lifetime risk of developing BC and OC by about 85% and 60%, respectively [32]. A germline mutation of the BRCA genes is the leading cause of about 5% of BCs [33, 34]. Studies also establish that the cumulative risk of contralateral BC is 40% and 26% for BRCA1 and BRCA2 carriers, respectively, after about 20 years of initial BC diagnosis [35]. A study on triple-negative breast cancer (TNBC) patients revealed that around 16.7% of patients studied have a deleterious mutation in the BRCA genes [36]. Another study on TNBC patients shows that BRCA1 genetic testing is appropriate for women who develop TNBC, regardless of family history of breast cancer or ovarian cancer [37]. Besides that, BRCA2 mutations increase the risk of developing breast cancer in males [38]. The prevalence of the BRCA1/2 variant may be higher than expected [39]. However, in the Beitsch study [29], it was shown that if genetic tests were performed only based on the NCCN Criteria, and when only the BRCA1/2 or limited panels were tested, about 45% of breast cancer patients with germline variants would be ignored. Buys et al. [40] genetically tested 35,000 breast cancer patients with a panel of 25 hereditary cancer genes. They found that 9.3% of participants had pathogenic variants. More than 50% of these variants were related to genes other than BRCA1/2, including CHEK2, ATM, and PALB2. A higher frequency of pathogenic variants was observed in women younger than 40 years of age [40].

A clinical cohort study evaluates the long-term effects of genetic testing for breast cancer and ovarian cancer predisposition. According to the study, compared with non-carriers, post-genetic testing carriers reported more risk management activities, including risk-reducing surgery, mammograms, and colorectal and prostate screening [38]. Graves and colleagues [6] studied 105 women with a negative breast biopsy and a breast cancer or ovarian cancer family history. They found that people who were more concerned about cancer and knew more about the benefits of genetic testing were more likely to have genetic testing. It is not much related to their actual risk based on family or personal history [6]. The availability of genetic information in patients with cancer is an excellent chance in the practice of medicine [29]. A population-based study reports patients’ willingness to receive genetic testing, and 29% of them have it. According to a report, approximately 50% of high-risk patients performed a genetic test. The primary reason for not testing high-risk patients was the lack of a physician’s recommendation, not the expense. Also, genetic counseling is done only for nearly 40% of all high-risk women [9]. Another study confirms that 50% to 80% of at-risk persons do not receive genetic testing because there are limitations in the use of family history, and insurance rarely covers such tests [29]. An updated statement on genetic testing accepted by the American Society of Breast Surgeons may facilitate routine referral of all patients diagnosed with BC for genetic study [41].

**Figure 3:**
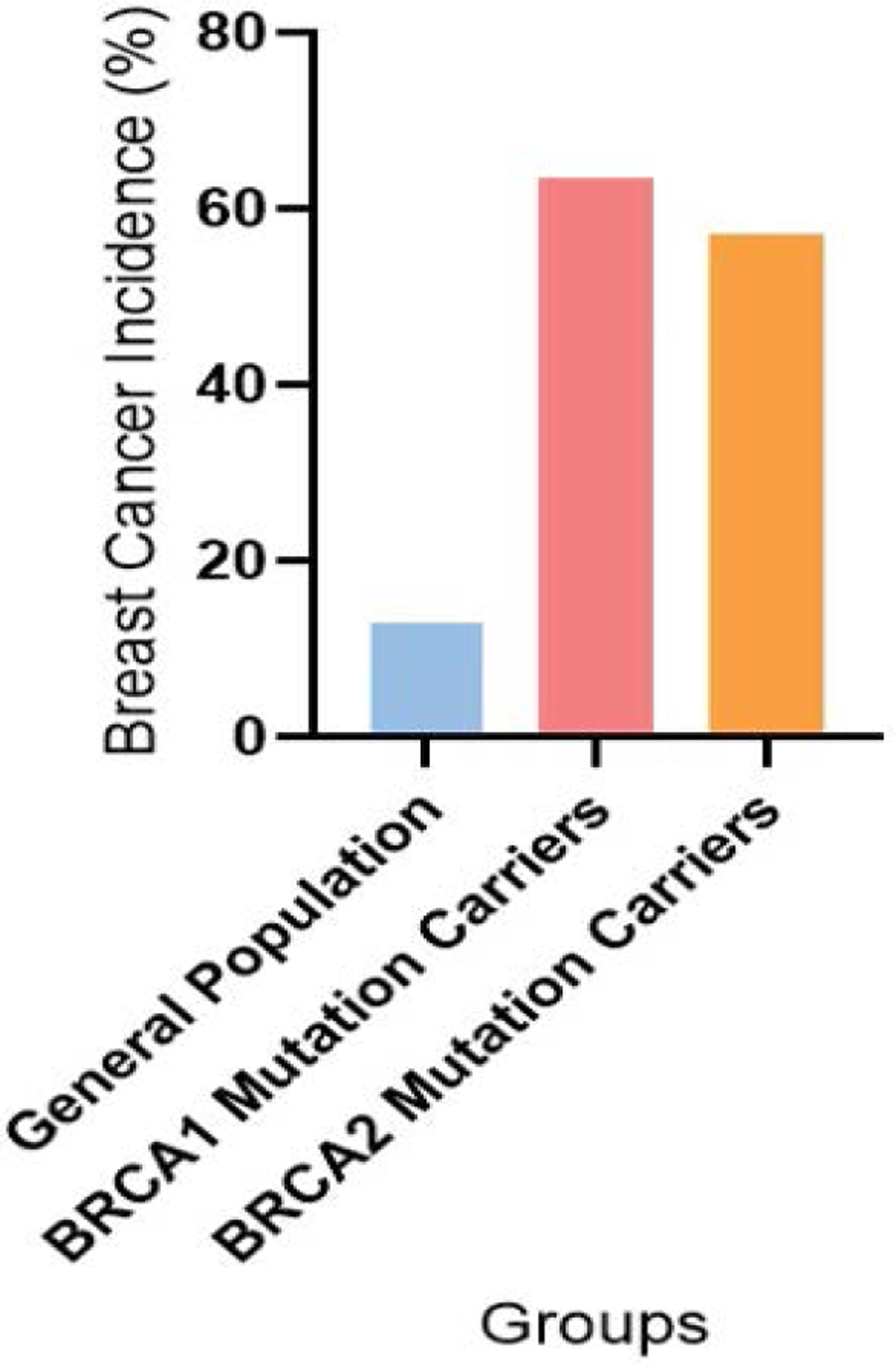
Incidence of breast cancer by BRCA mutation status in three different groups.

Figure 3 shows the incidence of breast cancer in various categories including General Population (13%), BRCA1 Mutation Carriers (55% to 72% with a mean of 63.5%), and BRCA2 Mutation Carriers (45% to 69% with a mean of 57%) [7,42,43,44].

**Figure 4:**
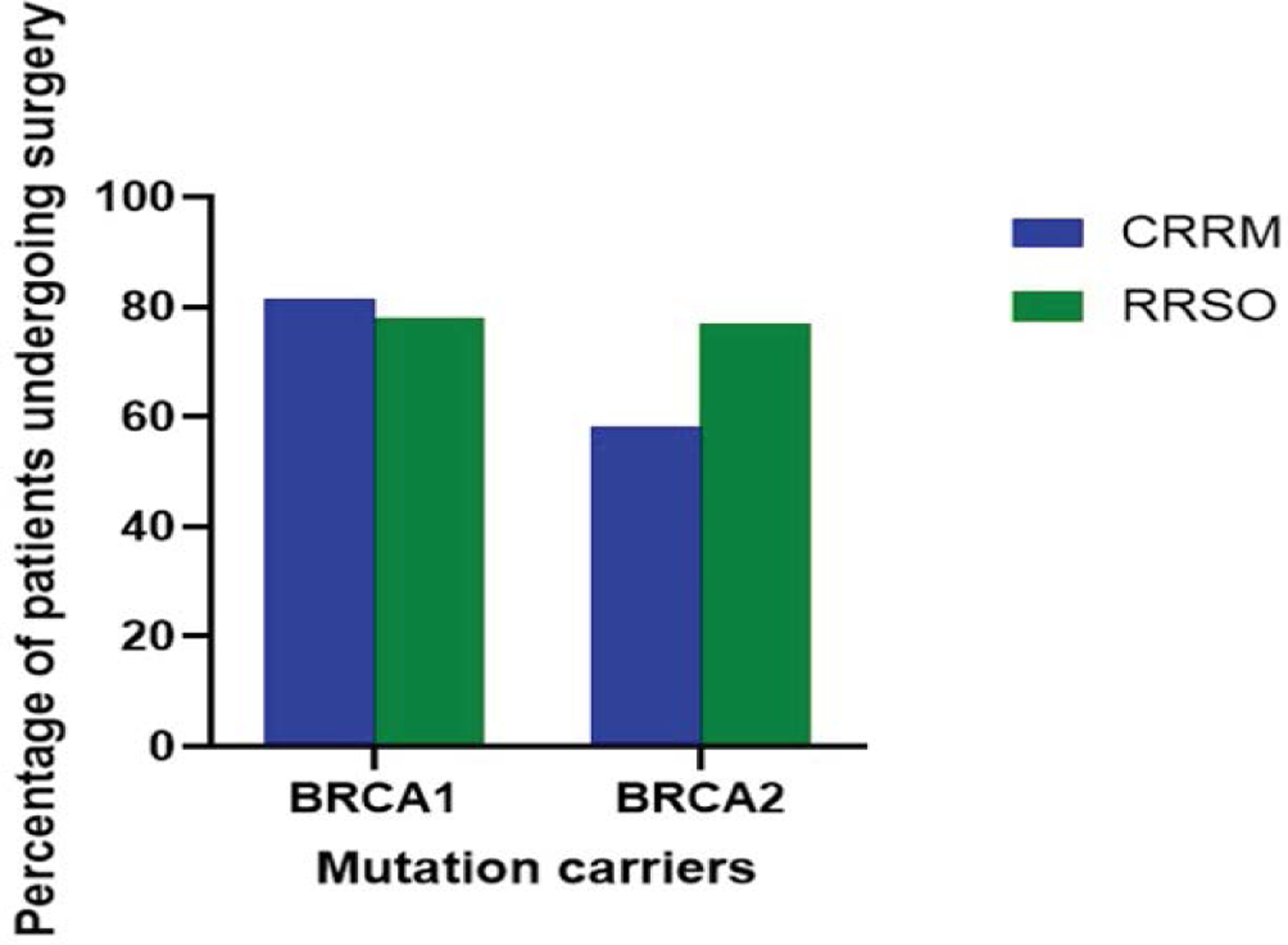
Percentage of patients undergoing surgery with BRCA1 and BRCA2 mutation carriers. CRRM, contralateral risk-reducing mastectomy; RRSO, risk-reducing salpingo-oophorectomy.

Figure 4: Percentage of patients undergoing surgery with BRCA1 and BRCA2 mutation carriers. CRRM, contralateral risk-reducing mastectomy; RRSO, risk-reducing salpingo-oophorectomy. As shown in Figure 4, 81.3% of BRCA1 and 58.1% of BRCA2 patients underwent a contralateral risk-reducing mastectomy (CRRM). 78.0% of BRCA1 and 76.9% of BRCA2 patients underwent risk-reducing salpingo-oophorectomy (RRSO). The bars in Figure 4 illustrate the significant reduction in the incidence of breast cancer among patients who underwent prophylactic mastectomy and salpingo-oophorectomy, compared to those who did not undergo these surgeries [45].

**Figure 5:**
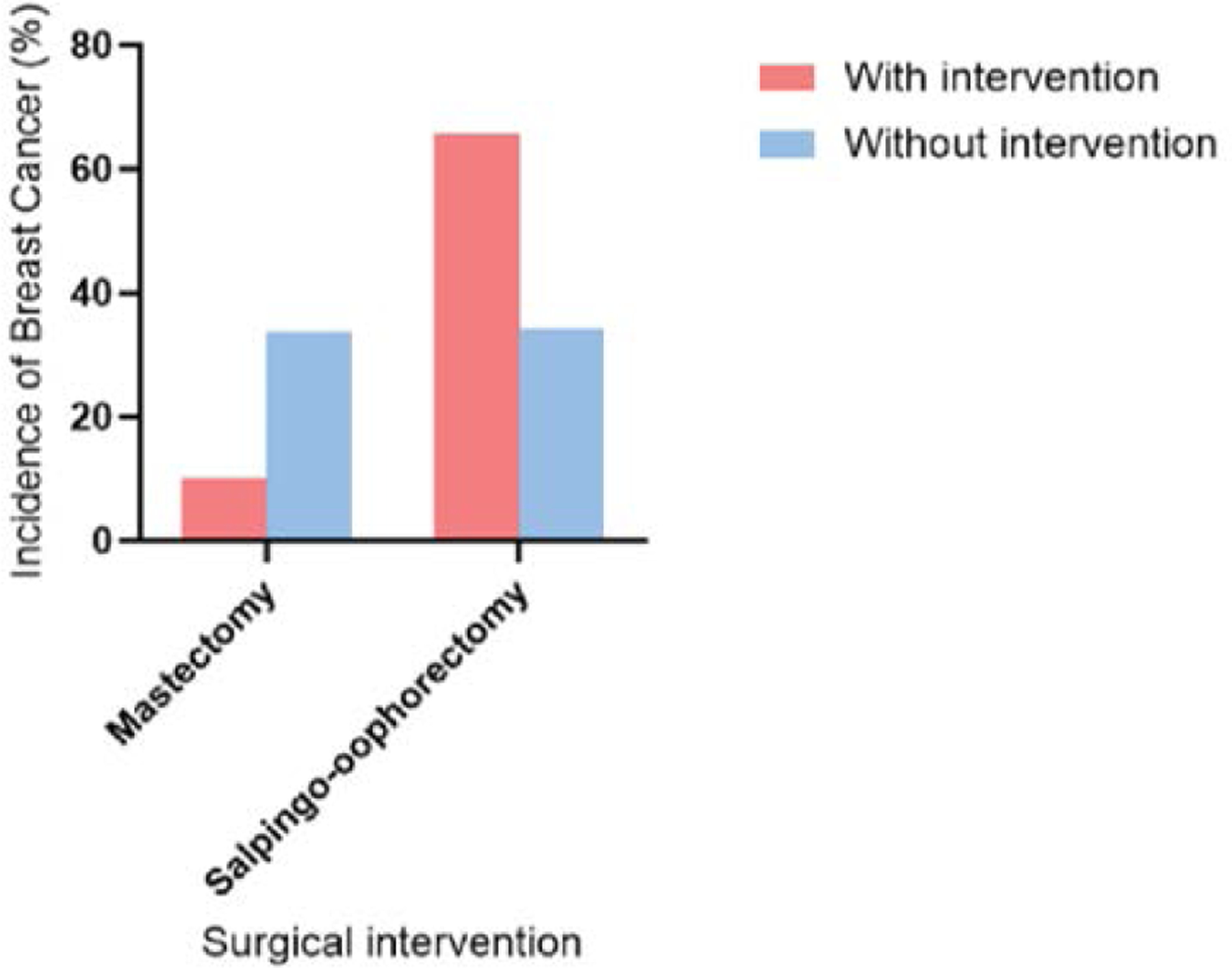
Impact of prophylactic surgery on breast cancer incidence. The data show mean percentage of incidence of breast cancer without and with two surgical intervention with mastectomy and salpingo-oophorectomy.

As shown in Figure 5, the incidence of breast cancer was significantly lower for those that underwent prophylactic mastectomy (10.2%) compared to those that did not (33.7%). However, it was observed that patients that underwent prophylactic salpingo-oophorectomy had an increased incidence of invasive disease (65.7%) compared to patients that did not (34.3) for those who underwent the procedures compared to those who did not (33.7% for no mastectomy and 34.3% for no salpingo-oophorectomy) [45].

## Prostate cancer

Prostate cancer (PCA) is the second most prevalent malignancy in men and the fifth cause of death worldwide [46]. Studies demonstrate that age, race, family history of PCA, and Comorbidity are important risk factors for developing the disease [47]. Approximately 5–10% of all PCA are hereditary. A first-degree relative with PCA increases the risk of the disease 2–3 times, related to hereditary mutations in high-, moderate-, and low-penetrance genes [20]. The most frequent screening test to identify prostate cancer is determining prostate-specific antigen (PSA) levels in the blood, but the positive predictive value is relatively low [20]. Besides, widespread mass screening may cause unnecessary biopsies, over-detection, and over-treatment in PCA patients. Therefore, new diagnostic strategies may efficiently identify high-risk persons and provide optimal screening for those men who benefit from closer monitoring or diagnostic biopsy and surgery before metastasis [36, 37]. Prostate cancer patients with BRCA2 mutations often respond better to platinum-based chemotherapy like carboplatin, since their tumors are more vulnerable to DNA repair–targeting drugs. [48]. Screening for HOXB13 is recommended for suspected hereditary PCA cases [20, 49]. Additionally, studies confirm the association between pathogenic variants in genes such as BRCA1/2, ATM, CHEK2, FANCA, PALB2, and PCA diagnosis [46].

**Figure 6:**
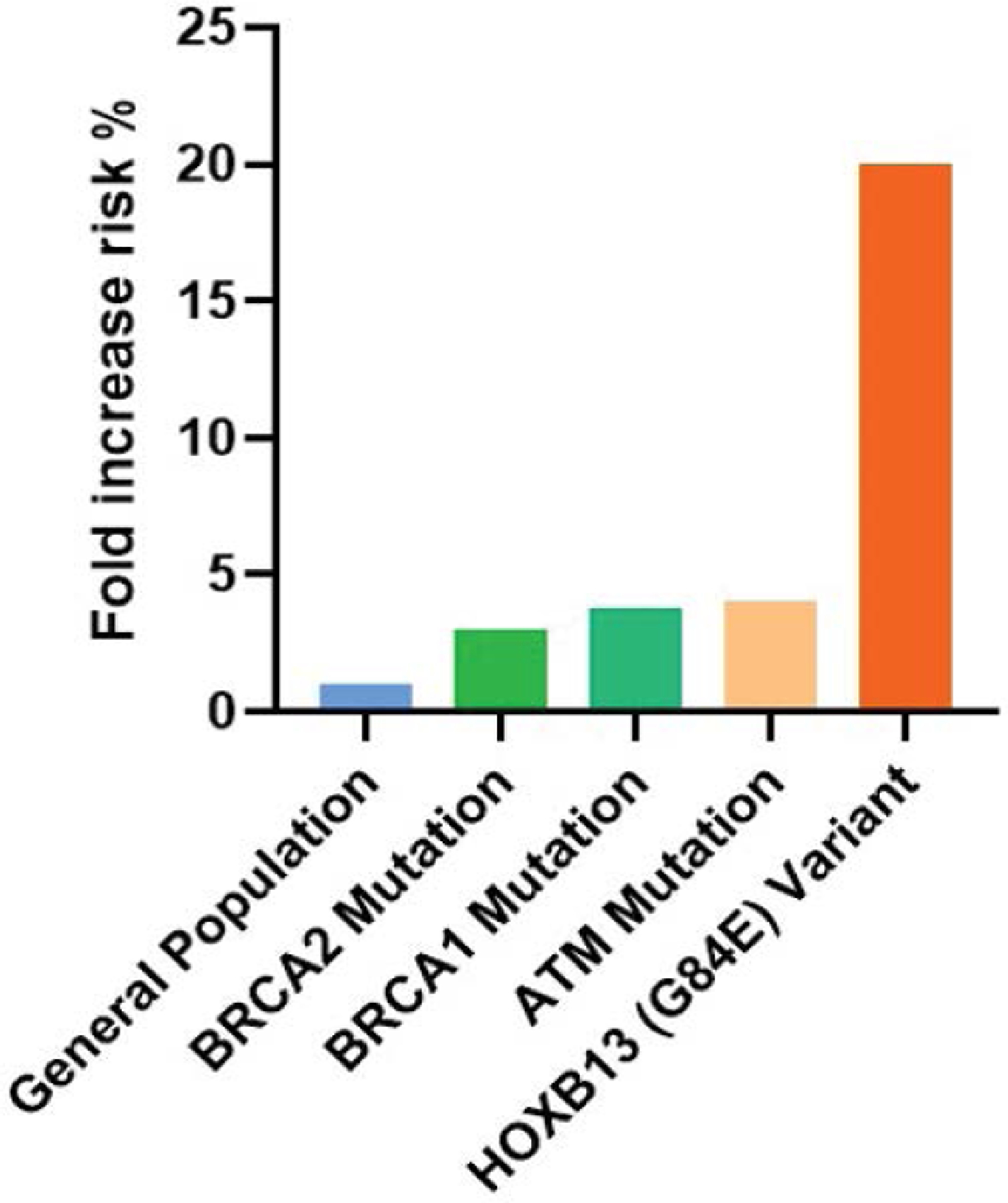
Risk Increase in Prostate Cancer due to Hereditary Mutations shown on the x-axis.

In Figure 6, the bars illustrate the relative risk of developing prostate cancer associated with specific genetic mutations compared to the general population. The data demonstrates significant risk increases for individuals carrying mutations in BRCA1, BRCA2, ATM, and HOXB13 genes, with the HOXB13 (G84E) variant showing the highest fold increase in risk. General Population: The baseline risk of prostate cancer for the general male population is set at 1x. This serves as a reference point against which the increased risks associated with genetic mutations are measured. BRCA2 Mutation: Men who carry a BRCA2 mutation have a threefold increased risk of developing prostate cancer compared to the general population. This mutation is associated with more aggressive forms of prostate cancer and earlier onset of the disease. BRCA1 Mutation: Individuals with a BRCA1 mutation have an approximately 3.75-fold higher risk of developing prostate cancer. Like BRCA2, this mutation also correlates with more aggressive cancer phenotypes and poorer outcomes. ATM Mutation: The ATM mutation is associated with a fourfold increased risk of prostate cancer. ATM is crucial for DNA repair, and mutations in this gene can lead to significant cellular abnormalities and cancer development. HOXB13 (G84E) Variant: This variant shows the most substantial increase in risk, with carriers having a 20-fold increased likelihood of developing hereditary prostate cancer. HOXB13 plays a significant role in prostate development, and its mutation can lead to early-onset and familial prostate cancer [50–57].

## Colorectal Cancer

Colorectal cancer (CRC) is a frequently diagnosed type of cancer among Americans, and about 145,600 CRC cases are diagnosed annually in the US [58]. At the global level, colon and rectal cancers represent the third most common form of cancer, and colon cancer is more common than rectal cancer [59]. CRC is a multifactorial disease with racial disparities in mortality rates [60], and individuals with a first-degree relative with the disease are 2 to 3 times more likely to develop CRC [61]. Studies establish that known genetic CRC syndromes are related to approximately 2% to 5% of all CRC cases. Without appropriate treatment, the lifetime risk of CRC significantly increases. Besides, 25% of all CRC cases happen to people with a family history of the disease and no known genetic disorders [7]. Early identification of at-risk people can provide a practical schedule for performing relevant diagnostic tests such as fecal occult blood tests, sigmoidoscopy, and colonoscopy at appropriate intervals [60]. Modifying dietary patterns and lifestyles, such as maintaining a healthy diet, limiting red meat consumption, taking supplements such as vitamin D, physical activity, and avoiding smoking and alcohol, can efficiently reduce colorectal cancer risk [62]. Several types of research reveal the role of genetic factors in providing insight into the prevention, prognosis, and treatment of colorectal cancers. For instance, the mutation in KRAS and B-RAF genes can affect the disease’s future treatment [63]. As mentioned before, hereditary syndromes are related to the development of colorectal cancer and are caused by germline mutations in genes such as APC, MYH, STK11/LKB1, BMPR1A, SMAD4, and DNA mismatch repair [64]. Similarly, a study identifies HFN4A, CHDH1, and LAMB1 among the genes associated with susceptibility to ulcerative colitis and CRC [65].

### Genetic Syndromes and CRC Risk:

(i) Lynch Syndrome (HNPCC): Lynch syndrome is the most common hereditary CRC syndrome, caused by mutations in DNA mismatch repair (MMR) genes such as MLH1, MSH2, MSH6, and PMS2. Individuals with Lynch syndrome have a significantly increased risk of developing CRC, often at a younger age compared to the general population. The risk of CRC in Lynch syndrome patients can be as high as 80% [61]. (ii) Familial Adenomatous Polyposis (FAP): FAP is caused by mutations in the APC gene and is characterized by the development of hundreds to thousands of polyps in the colon and rectum during adolescence. If left untreated, nearly all individuals with FAP will develop CRC by age 40 [66]. (iii) MUTYH-Associated Polyposis (MAP): MAP results from biallelic mutations in the MUTYH gene, leading to a high lifetime risk of CRC, though generally lower than that seen in FAP [61]. (iv) Peutz-Jeghers Syndrome: This syndrome is caused by mutations in the STK11 gene and is associated with hamartomatous polyps in the gastrointestinal tract and an increased risk of CRC [67].

### Regular Screening:

For individuals with a known genetic predisposition, regular screening such as colonoscopies can significantly reduce CRC mortality. High-risk individuals often begin screening at a younger age and undergo more frequent testing [63]. Prophylactic Surgery: In high-risk populations, prophylactic colectomies can be considered to prevent CRC development. This is particularly relevant for individuals with FAP and other high-risk syndromes [68]. Regarding lifestyle modifications, adoption of a healthy lifestyle, including a diet rich in fruits, vegetables, and whole grains, regular physical activity, and avoidance of smoking and excessive alcohol intake, can reduce CRC risk. Additionally, certain dietary supplements such as vitamin D have been suggested to have a protective effect against CRC [69].

**Figure 7:**
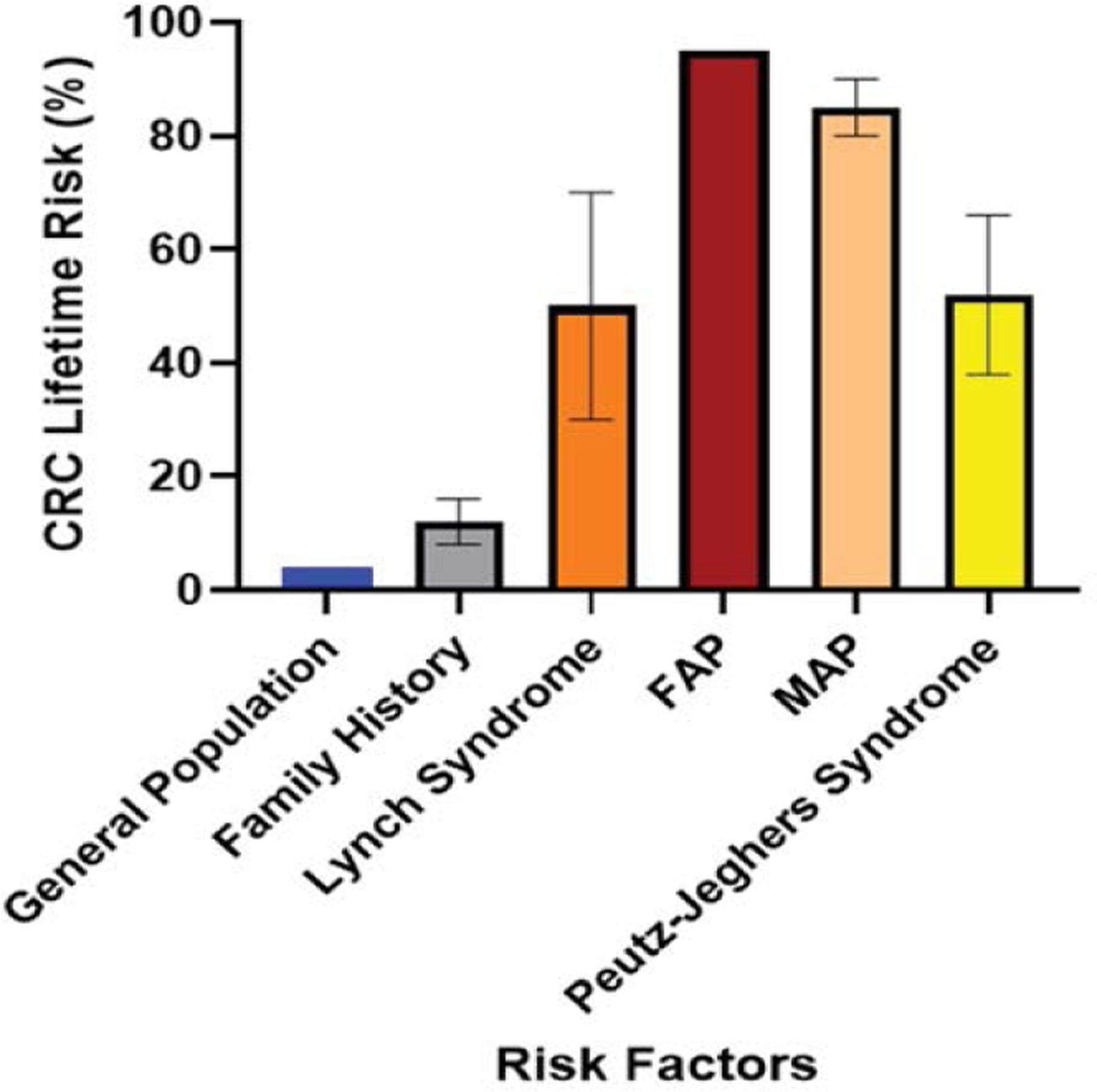
This bar chart illustrates the lifetime risk of developing colorectal cancer (CRC) among different groups based on genetic conditions.

In Figure 7, the general population has a 4% risk, which increases two to four-fold or individuals with a family history of CRC (8–16%). Those with Lynch Syndrome have a 30–70% lifetime risk, while Familial Adenomatous Polyposis (FAP) carriers have a 95% risk. The risk for MUTYH-associated polyposis (MAP) carriers is 80–90%, and for those with Peutz-Jeghers Syndrome, it is 38–66%. The variances for each risk category are shown with error bars [61,67, 69–73].

**Figure 8:**
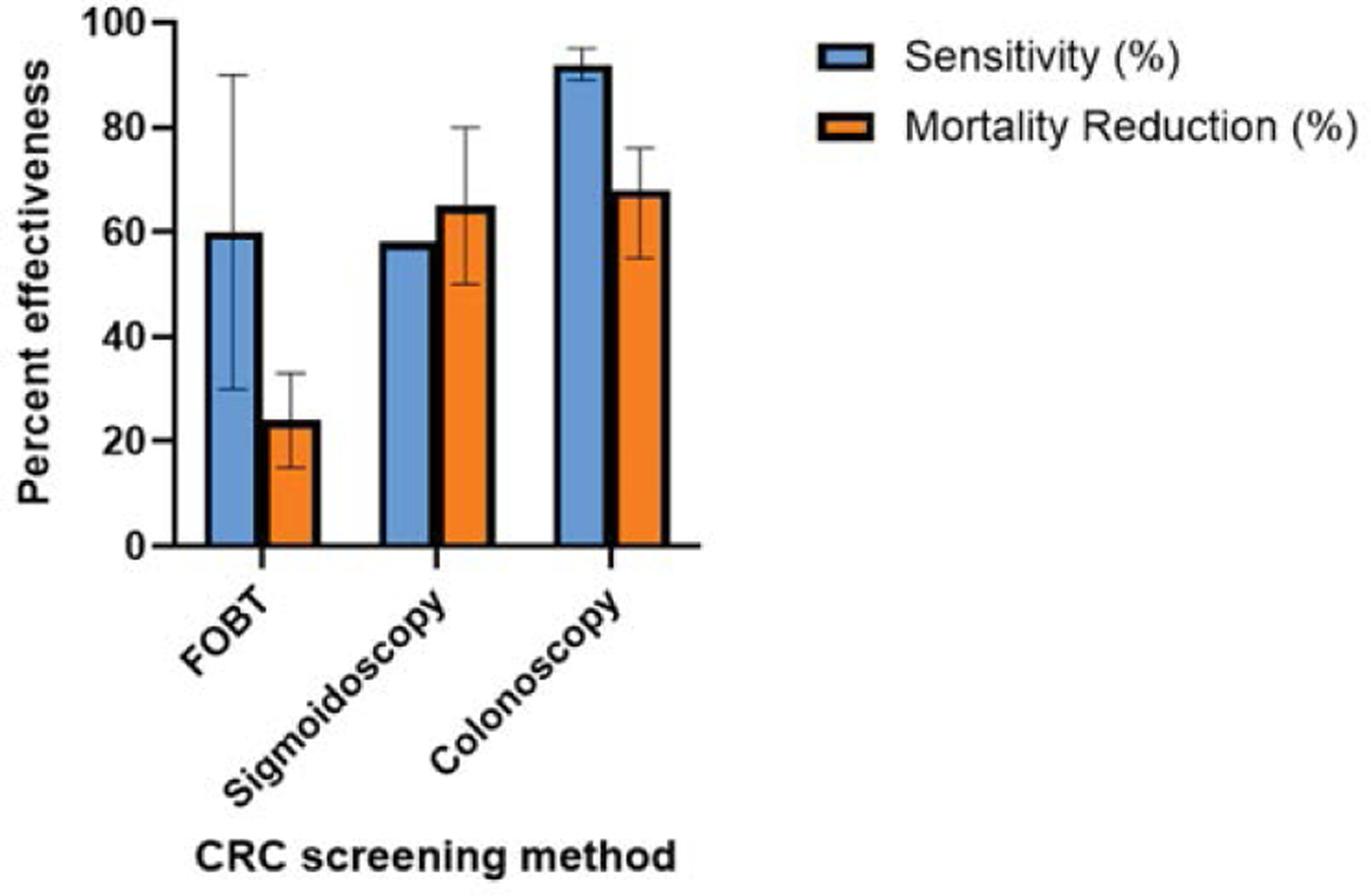
Effectiveness of colorectal cancer screening methods. Percent sensitivity (in blue color) and percent mortality reduction (in orange color) are provided. The variation in the range of percent in various studies is shown by lines within each bar. FOBT, fetal occult blood testing.

Figure 8: Effectiveness of colorectal cancer screening methods. Percent sensitivity (in blue color) and percent mortality reduction (in orange color) are provided. The variation in the range of percent in various studies is shown by lines within each bar. FOBT, fetal occult blood testing.

The data in Figure 8 show the comparison of the effectiveness of various colorectal cancer screening methods. The blue bars represent the sensitivity rates, with fecal occult blood testing (FOBT) between 30–90%, sigmoidoscopy 58%, and colonoscopy at 89–95%. The orange bar indicates the mortality reduction rates with FOBT between 15–33%, sigmoidoscopy at 50–80% and colonoscopy at 55–76%, [52,56,74–76]. The percent range for each screening method are shown with error bars. Table 2 demonstrates outcome of prophylactic procedures in CRC development and survival [77–78].

## Pancreatic Cancer

Pancreatic ductal adenocarcinoma (PDA) is one of the deadliest cancers worldwide, which accounts for 95% of all pancreatic cancer cases. PDA is anticipated to become the second most prevalent cause of cancer-related deaths in the US by the next decade [76,79]. PDA is diagnosed in more than half of the patients in a late stage, which reduces their chances of being alive five years after they were diagnosed to 2% [79]. Although stroma depletion has been suggested as a therapeutic intervention in addressing PDA, recent studies dispute and illustrate that stroma targeting leads to tumor amplification, leaving chemotherapy a better solution [80]. Further studies have suggested radiotherapy as an intervention using hypofractionated photons constituting high photo-doses to elicit an immune response and cell lysis [81]. Nevertheless, pancreatic cancer is mainly resistant to present treatment methods, and there is a need for new approaches to providing helpful information to increase prognosis and improve the treatment of patients with this disease [82].

The leading cause of PDA condition is not yet established; however, several hereditary factors have been identified to be shared among participants suffering from PDA, who have been clinically studied that are worth screening for preventative care [83]. Studies have established a significant increase in the chance of PDA diagnosis for individuals with immediate family members with pancreatic cancer and hereditary cancer syndromes [84,85]. For instance, Familial Atypical Multiple Mole Melanoma Syndrome (FAMMM) and pancreatic intraepithelial neoplasias (PanINs) are related to mutations of CDKN2A and K-RAS, respectively, and correlated with PDA development [86, 87]. Besides, mutations in ATM, BRCA1, BRCA2, CHEK2, and PALB2 are associated with Familial PC. [86]. Similarly, studies show that patients with BRCA and PALB2 germline mutations are more likely to respond to platinum-based chemotherapy than carriers of other mutations. [88]. The penetrance of PDA in individuals with familial pancreatic cancer may increase due to environmental and behavioral risk factors, such as smoking [89]. Consequently, genetic screening can effectively offer the best preventive and treatment strategy for mutation carriers.

Table 3 shows the mortality rates within five years of diagnosis for patients with mutations in the BRCA1, BRCA2, ATM, CHEK2, and PALB2 genes compared to those without these mutations. Studies have demonstrated that patients with these genetic mutations have different prognoses and responses to treatment [84–89].

**Figure 9:**
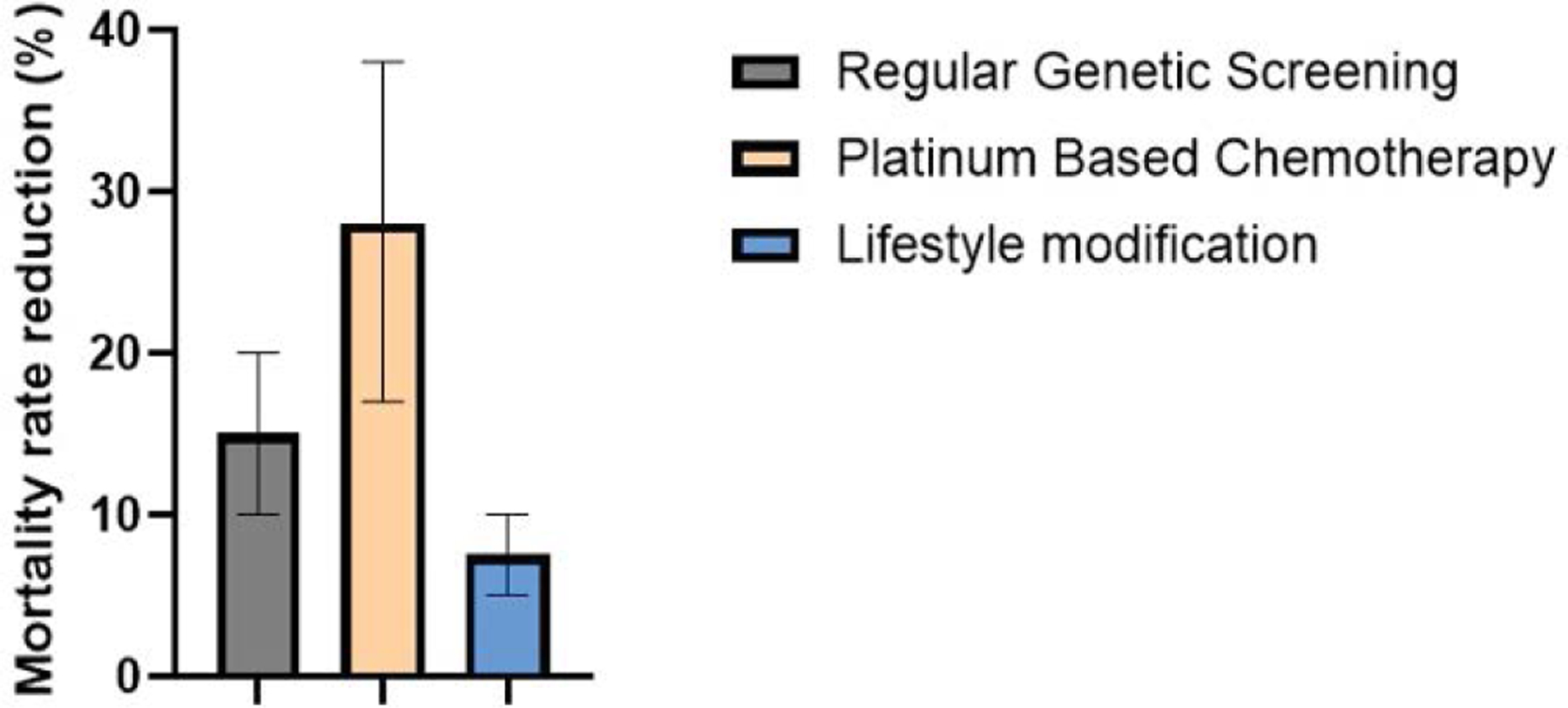
Efficacy of Preventive Care and Testing in Reducing Mortality Rates of Pancreatic Ductal Adenocarcinoma.

The data in Figure 9 demonstrates the median effectiveness of various preventative care and genetic testing strategies in reducing mortality rates among patients at high risk for pancreatic ductal adenocarcinoma (PDA) with error bars indicating percentage range. It compares the mortality rates of individuals undergoing regular genetic screening (10–20%), those receiving platinum-based chemotherapy based on their genetic profile (17–38%), and those adopting lifestyle modifications such as smoking cessation (5–10%). The data underscores the importance of early detection and personalized treatment strategies in improving patient survival outcomes [90].

## Uterine cancer

Uterine cancer ranks as the fourth most common cancer among women in the US [91]. Uterine cancer is another term for endometrial cancer due to its high prevalence within the diagnosis of uterine cancer. Endometrial cancer refers to the formation and thickening of the uterus lining in women, which usually results from the imbalance of circulating estrogen after menopause [92]. Studies have further demonstrated the thickening of the uterus lining caused by increased estrogen levels in association with progesterone [93]. In addition to the imbalance of hormones and other significant environmental factors such as obesity, estrogen-inducing medication, exercise, and diet, genomic and hereditary factors have also been demonstrated to affect uterine cancer diagnosis significantly [94]. For instance, Lynch syndrome and hereditary non-polyposis colon cancer are associated with MMR gene mutations and increase the risk of endometrial, colon, and ovarian cancer [95]. Studies establish that women with a history of first-degree relatives with endometrial cancer have a higher chance (RR of 1.82) of developing the disease [96].

**Figure 10:**
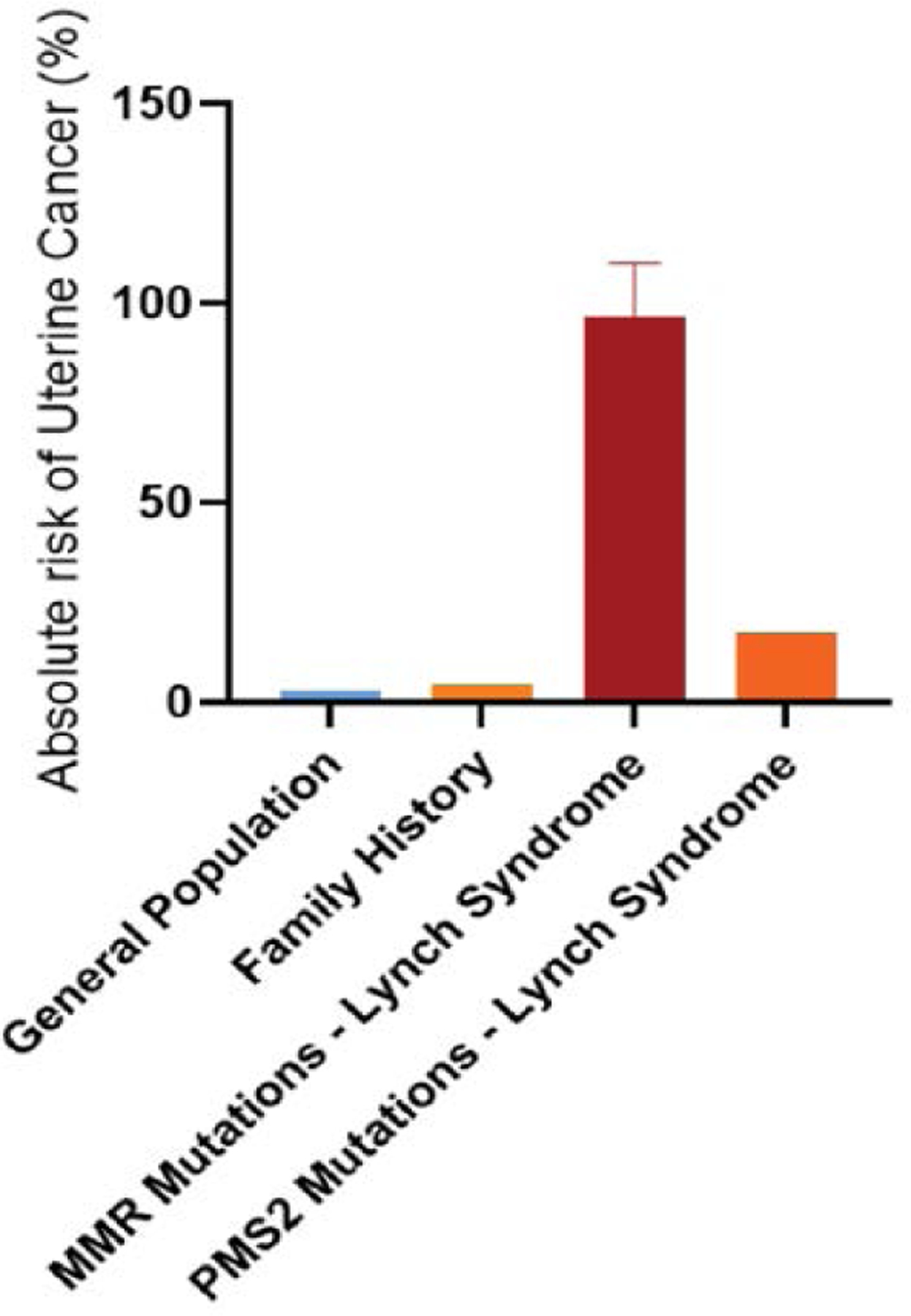
Absolute Risk of Uterine Cancer Based on Genetic Mutations.

The bar graph in Figure 10 illustrates the absolute risk of uterine cancer among the general population (3.1%), women with a family history of uterine cancer (4.34%), women with Lynch Syndrome with MMR gene mutations (82.7–110%), and women with Lynch syndrome with PMS2 mutations (17.7%) Absolute risk was calculated by estimating the product between the absolute risk of the general population and the reported odds ratio/cumulative incidences/relative risk of the reported studies. The graph highlights the increased risk associated with genetic mutations and family history, emphasizing the need for targeted screening and preventative strategies in high-risk groups [97–99].

Table 4 shows a comparison on the risk reduction rates of various preventive measures in reducing the risk of uterine cancer. The measures include progesterone therapy, lifestyle modifications (such as diet and exercise), and adjustments to hormone replacement therapy. The graph highlights the importance of preventive care in managing and reducing the risk of uterine cancer [99–106].

## Conclusion

Predictive genetic testing represents a transformative approach in the landscape of oncology, enabling the early detection and precise treatment of cancer through genetic insights. This review underscores the significance of identifying genetic mutations in asymptomatic individuals, particularly those with a family history of genetic diseases [2]. Techniques like next-generation sequencing (NGS) have revolutionized our capability to analyze multiple genes simultaneously, thereby enhancing our understanding of cancer predisposition and tailoring prevention strategies to mitigate risk effectively [107, 108]. The application of predictive genetic testing extends beyond risk assessment, influencing the development of individualized therapeutic strategies that significantly reduce the morbidity and mortality associated with genetic disorders. In cases such as breast and ovarian cancer, the detection of BRCA1 and BRCA2 mutations facilitates targeted interventions, including chemoprevention and risk-reducing surgeries, which have proven efficacy in lowering the incidence of cancer in high-risk populations [2, 109, 110]. Furthermore, the integration of advanced technologies like targeted nanoparticle platforms exemplifies the next frontier in cancer treatment. The study by [111], where a novel cancer-targeting peptide-functionalized nanoparticle using gold nanoparticles and Thioctic acid-DMPGTVLP peptide conjugate was developed, highlights the potential of nanomedicine in overcoming the traditional challenges of drug delivery and resistance in cancer therapy. These nanoparticles, characterized by their selective affinity and enhanced cellular uptake, induce apoptosis more efficiently in cancer cells than conventional treatments, suggesting a promising avenue for future cancer therapeutics [111]. Despite these advancements, the field of predictive genetic testing faces ongoing challenges, including ethical considerations, variable expressivity, and the psychological impact of genetic information. However, the evolving landscape of genetic testing and molecular diagnostics holds promise for a more profound understanding and management of hereditary cancers [1, 3]. The continuous refinement of genetic testing methodologies and the integration of novel therapeutic modalities, such as the receptor-specific peptide-gold nanoparticle platforms, will significantly enhance our ability to manage and treat cancer more effectively, heralding a new era of precision oncology.

## Figures and Tables

**Table 1:** Studies detailing the percentage of germline mutations in ovarian cancer

Study Citation	Study Type	Population	Cohort size	Percentage Germline Mutations
Imterat et al (2023) [21]	Retrospective cohort	Germany	702	23.6
Aslop et al (2012) [22]	Population-based,	Australia	1,001	14.1
	Case-control			
Zhang et al (2011) [23]	Population-based,	Canada	1,342	13.3
	observational cohort			
Cotrim et al (2019) [24]	Retrospective cohort	Brazil	158	20.8
Norquist et al (2016) [25]	Population-based	United States	1,915	18
	observational cohort			
Paradiso et al (2019) [26]	Population-based/Hospital based	United Kingdom	2,222	8.1
	**Average percentage of germline mutations**			16.2 ± 5.1

**Table 2:** Outcomes of prophylactic surgery in colorectal cancer

Gene/Marker	Prophylactic Surgery	Outcome	Study
FAP	Total colectomy with ileorectal anastomosis (IRA) or proctocolectomy with ileoanal anastomosis (IAA) and J pouch	25-year overall survival of 97.8 % in the IRA group and 98.8 % in the IAA group	Pasquer A et al. [77]
MAP	Colectomy +IRA	4.3% developed CRC in follow up	Patel R et al. [78]

CRC, colorectal cancer; FAP, familial adenomatous polyposis; IAA, ileoanal anastomosis; IRA, ileorectal anastomosis; MAP, MUTYH-associated polyposis

**Table 3:** The impact of hereditary factors on patient outcomes among patients with pancreatic ductal adenocarcinoma (PDA) based on specific genetic mutations

Gene/Marker	Median overall survival (OS)	Estimated 5-Year survival	Compared to Baseline
BRCA1/2	27–45 mo	15–25%	Lower mortality
ATM	29–33 mo	~15%	Slightly lower
PALB2	20–24 mo	~10–15%	No difference
CHEK2	20–22 mo	~10%	No difference
No mutation	19–22 mo	7–10%	Baseline

**Table 4:** Effectiveness of Preventive Measures in Reducing Uterine Cancer Risk.

Preventative measure	Risk Ratio (RR)	Relative Risk Reduction	Study
Levongestrel IUD (Progesterone Therapy)	0.22	78%	Jareid et al. (2018) [100]
Continuous Estrogen plus Progestin	0.59	41%	Chlebowski et al. (2015) [101]
Unopposed Estrogen	1.79	−79%	Beral et al. (2005) [102]
Prophylactic Hysterectomy	0.00	100%	Schmeler et al. (2006) [103]
Intentional Weight loss >5%	0.61	39%	Luo et al. (2017) [104]
Physical Activity	0.80	20%	Moore et al. (2010) [105]
High Fiber Diet	1.00	0%	Cui et al. (2011) [106]
